# Dynamic simulation of motion effects in IMAT lung SBRT

**DOI:** 10.1186/s13014-014-0225-3

**Published:** 2014-11-01

**Authors:** Wei Zou, Lingshu Yin, Jiajian Shen, Michael N Corradetti, Maura Kirk, Reshma Munbodh, Penny Fang, Salma K Jabbour, Charles B Simone, Ning J Yue, Ramesh Rengan, Boon-Keng Kevin Teo

**Affiliations:** Department of Radiation Oncology, Rutgers Cancer Institute of New Jersey, Rutgers, The State University of New Jersey, New Brunswick, NJ 08903 USA; Department of Radiation Oncology, University of Pennsylvania, Philadelphia, PA 19104 USA; Department of Radiation Oncology, Mayo Clinic, Phoenix, Arizona 85054 USA; Department of Radiation Oncology, Dana-Farber Cancer Institute/Brigham and Women’s Hospital, Boston, MA 02115 USA; Department of Radiation Oncology, University of Washington, Seattle, WA 98195 USA

**Keywords:** SBRT, Lung motion, Interplay, IMAT

## Abstract

**Background:**

Intensity modulated arc therapy (IMAT) has been widely adopted for Stereotactic Body Radiotherapy (SBRT) for lung cancer. While treatment dose is optimized and calculated on a static Computed Tomography (CT) image, the effect of the interplay between the target and linac multi-leaf collimator (MLC) motion is not well described and may result in deviations between delivered and planned dose. In this study, we investigated the dosimetric consequences of the inter-play effect on target and organs at risk (OAR) by simulating dynamic dose delivery using dynamic CT datasets.

**Methods:**

Fifteen stage I non-small cell lung cancer (NSCLC) patients with greater than 10 mm tumor motion treated with SBRT in 4 fractions to a dose of 50 Gy were retrospectively analyzed for this study. Each IMAT plan was initially optimized using two arcs. Simulated dynamic delivery was performed by associating the MLC leaf position, gantry angle and delivered beam monitor units (MUs) for each control point with different respiratory phases of the 4D-CT using machine delivery log files containing time stamps of the control points. Dose maps associated with each phase of the 4D-CT dose were calculated in the treatment planning system and accumulated using deformable image registration onto the exhale phase of the 4D-CT. The original IMAT plans were recalculated on the exhale phase of the CT for comparison with the dynamic simulation.

**Results:**

The dose coverage of the PTV showed negligible variation between the static and dynamic simulation. There was less than 1.5% difference in PTV V95% and V90%. The average inter-fraction and cumulative dosimetric effects among all the patients were less than 0.5% for PTV V95% and V90% coverage and 0.8 Gy for the OARs. However, in patients where target is close to the organs, large variations were observed on great vessels and bronchus for as much as 4.9 Gy and 7.8 Gy.

**Conclusions:**

Limited variation in target dose coverage and OAR constraints were seen for each SBRT fraction as well as over all four fractions. Large dose variations were observed on critical organs in patients where these organs were closer to the target.

## Introduction

Stereotactic body radiation therapy (SBRT) has been increasingly employed in the treatment of medically inoperable early stage non-small cell lung cancer (NSCLC). SBRT involves hypofractionation to deliver a large dose per fraction in a small number (usually 1–5) of treatments to the target volume while minimizing normal tissue exposure. Studies have suggested that SBRT provides high rates of local control with few high grade toxicities [1]. Treatment plans are designed to provide sharp dose fall-off outside the target to avoid normal tissue toxicity. Intensity modulated arc therapy (IMAT) is a newer treatment modality that can deliver highly conformal dose distributions with fewer monitor units than intensity modulated radiation therapy (IMRT) [2]. IMAT delivers an optimized plan comprising of one or multiple arcs with continuous gantry rotation while modulating the fluence with the multi-leaf collimator (MLC) [3]. It is now widely adopted for lung SBRT [4] due to the reduced treatment time compared with IMRT and 3D-conformal treatment [5-8].

For the treatment of lung and abdominal tumors, the effect of respiratory motion on both the target and organs at risk (OAR) is a source of concern. Multiple methods that have been proposed to manage tumor motion include tumor tracking, breathing control, forced shallow breathing and gated delivery [9]. One way to account for tumor motion [10,11] is to create margins using an internal target volume (ITV) that encompasses the motion envelope of the tumor derived from a respiratory-gated computed tomography (4D-CT) [12]. During IMRT and IMAT delivery, the interplay between the target and the multi-leaf collimator motion can lead to dose discrepancies in conventional fractionation scheme of about two Gy per fraction. Studies [13-16] have shown that under multiple fields and after a large number of fractions, the interplay effect in IMRT delivery results in a smeared dose distribution where the standard deviation of the dose is generally within 1% of the expected value [13].

In contrast to traditional fractionation, the dose averaging effect in SBRT IMAT delivery using hypofractionation is expected to be smaller given the limited number of fractions. However, limited data exist assessing the dosimetric consequence from the interplay among the fluence, gantry and MLC motion. Experimental studies have been performed to examine the impact of this interplay effect using film dosimetry and phantoms [17-19]. Patient-specific study is desirable and can be accomplished using computer simulation of the interplay effect. Kuo *et al.* [20] studied a single arc therapy on hepatic cancer patients by warping the dose distribution of multiple 4D CT phases to a reference CT image using deformable image registration. Rao *et al.* [21] studied volumetric-modulated arc therapy (VMAT) using a single arc for a three fraction SBRT treatment regimen (60 Gy total) on a 80-leaf MLC Elekta linac (Elekta AB, Stockholm, Sweden) for lung cancer. In both cases, less than 1% difference was found on target dose distribution between the 4D calculation and the 3D calculation. In this study, we investigated the interplay effect on a four fraction IMAT RapidArc SBRT lung cancer treatment regimen (50 Gy total) using two arcs on a Varian linac with a 120-leaf MLC (Varian Medical Systems, Palo Alto, CA). Machine delivery data files were used to correlate the respiratory phase with the plan control points. Using 4D CT and deformable image registration tools, dynamic simulation of lung SBRT treatments was used to investigate the cumulative as well as inter-fraction dose deviations on target and organ at risk (OAR) dose distributions.

## Methods and materials

### Patient selection and simulation

Fifteen lung cancer patients with stage I peripheral tumors treated with SBRT were retrospectively selected for this study approved by the Institutional Review Board (University of Pennsylvania). Patients were simulated on either a Philips Gemini Big Bore PET/CT scanner (Philips Healthcare, Andover, MA) or a Siemens Sensation Open CT scanner (Siemens Medical Solutions USA, Inc. Malvern, PA). Both scanners were equipped with the Varian Real-time Position Management (RPM) system (Varian Medical Systems, Palo Alto, CA) for 4D scans. The CT images acquired included a free-breathing CT and a 4D CT image set. Abdominal compression was not employed. The target motion throughout the respiratory cycle was analyzed in three directions: superior-inferior, left-right, and anterior-posterior. As the interplay effect is expected to potentially have a larger effect on the delivered dose when there is large motion, only patients with >10 mm GTV motion in the superior-inferior direction were selected for this study. The mean motion of this cohort of patients was 12.9 mm with 2.7 mm standard deviation.

### 3D RapidArc plans

The initial patient RapidArc plans were optimized using the Eclipse treatment planning system (Varian Medical Systems, Palo Alto, CA). The GTV was determined on each respiratory phase of the 4D CT and used to generate the ITV that enclosed all of the identified GTVs from each respiratory phase. A 3 mm uniform expansion margin was applied to the ITV to obtain the PTV. The RapidArc plans utilized two coplanar arcs with reciprocal end points typically spanning approximately 200 degrees around the ipsilateral lung and a dose of 50 Gy in four fractions was delivered to the PTV. The plans were optimized on the free-breathing CT image in Eclipse and the anisotropic analytical algorithm (AAA) was used to calculate dose. These plans met the PTV and OAR constraints that were adopted from RTOG 0915 [22].

### 4D RapidArc plan partitioning

In order to simulate respiratory motion effects on RapidArc beam delivery, the plan control points which define the gantry angle, MLC leaf position, MUs of the original plan have to be associated with the respiratory phase. These control points were optimized to achieve the desired dose distribution while satisfying the machine hardware constraints [22]. As each arc of a SBRT plan was delivered through several breathing periods (range 2.2-4.3 seconds), association of control points to the respiratory phase of a 4D CT was made by making the assumption that patient specific respiratory cycle, as measured during the 4D CT, was periodic and representative of patient respiration during RapidArc delivery. Using the timestamps within the Varian linac Dynalog files which recorded the MLC leaf positions and gantry angle at every 50 ms time interval during beam delivery, each arc was partitioned into a series of smaller sub-arcs that contained only beams that were correlated to a particular respiratory phase.

**Figure 1:**
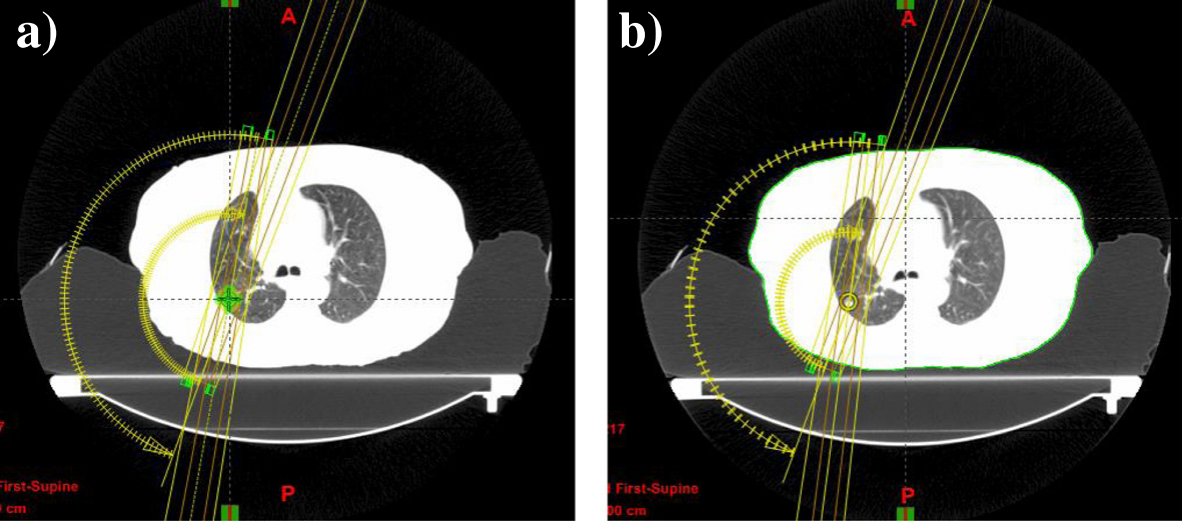
**Original and partitioned RapidArc plans. a)** The original RapidArc plan and corresponding control points indicated by short lines along the arcs. **b)** A partitioned RapidArc plan with a subset of original control points and interpolated control points associated with one respiratory phase. This partial plan is used to compute the dose delivered to the patient at one respiratory phase.

The control points of each arc can be expressed mathematically as

$$ {C}_{org}=\kern0.5em \left\{{C}_i:{\theta}_i, ML{C}_{i,j},M{U}_i,{t}_i\right\},\kern0.5em {C}_i\in {I}_0 $$

where *I*_*0*_ is the collection of control points in the original plan; *θ*_*i*_ is the gantry angle at *i*th control point, *MLC*_*i,j*_ is the *j*th leaf sequence of the two MLC banks, *MU*_*i*_ is the delivered MU at the *i*th control point; *t*_*i*_ is the time from the start of the arc delivery to the time the *C*_*i*_ control point is executed. The time values were extracted from the patient delivery Dynalog files. These control points were indicated as short lines along the original arcs in Figure 1a as an example. To associate the arc with the breathing cycles, the arc was first divided into time intervals *Δt = T*_*R*_*/N* where *T*_*R*_ is the patient respiratory period and *N* is the number of breathing phases used. Representatively four (*N* = 4) respiratory phases, namely, the 0% (maximum inhalation), 25%, 50% (maximum exhalation), and 75%, were adopted in this study. Additional control points at the transition between respiratory phases were inserted into the arc to represent the exact start and end points of each respiratory phase. The parameters of these additional control points were derived from time-based linear interpolation of the two adjacent original control points. The total control points then consisted of a collection of the original and interpolated control points,

$$ {C}_{tot}=\kern0.5em \left\{{C}_i:{\theta}_i, ML{C}_{i,j},M{U}_i,{t}_i\right\},\kern0.5em {C}_i\in I\hbox{'}, $$

where *I’* is the collection of the original and the additional control points. This cohort of control points *C*_*tot*_ were then partitioned and reassembled into four sub-arcs that were associated with each of the four respiratory phases,

$$ {C}_{tot\_n}=\kern0.5em \left\{\;{C}_i:{\theta}_i, ML{C}_{i,j},M{U}_i,{t}_i, at\kern0.5em {t}_i\in \left[M\cdot {T}_R+\left(n-1\right){T}_R/N,M\cdot {T}_R+n{T}_R/N\right]\right\},\kern0.5em {C}_i\in I\hbox{'},n=1\dots N $$

where *M* is an integer representing the number of respiratory cycles that have elapsed during the delivery of the arc. Control points that were outside of the corresponding respiratory phase had zero MUs and were excluded. An example of a partitioned plan associated with one CT phase was shown in Figure 1b. Plan partitioning was performed using MATLAB (MathWorks, Inc. Natick, MA) software and re-imported into the treatment planning system for 4D dose calculation.

### 4D Dose calculation

#### D.1. Respiratory phase initialization and 4D dose accumulation

**Figure 2:**
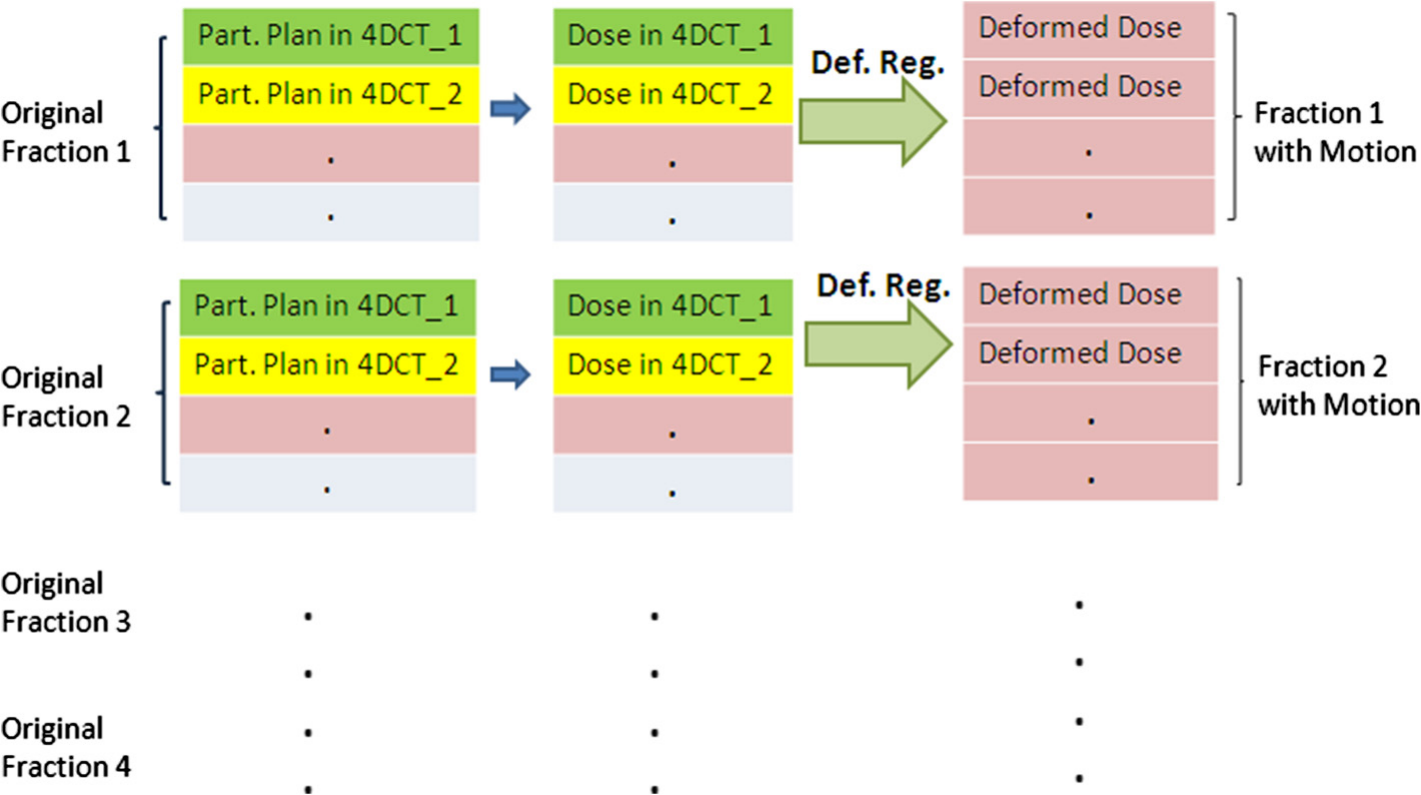
**Flow chart of plan partitioning, dose calculation and dose accumulation using 4D CT images.** Here “Part. Plan” represents “Partitioned Plan”, “Def. Reg.” represents “Deformable Registration”.

A random initial respiratory phase *n*_*0_k*_ was selected for each arc in fraction *k*. The dose delivered to each respiratory phase *n* at each fraction *D*_*n,n0_k*_ was forward calculated using the partitioned plans on the associated phase of the 4D CT. Figure 2 showed a flow chart illustrating the process. The maximum exhalation phase (50%) was chosen as the reference CT phase for dose accumulation. Each phase of the 4D CT image set was registered to the reference CT using MIM Maestro (MIM Software Inc., Cleveland, OH) deformable image registration software. The deformation vector fields from the source to target images can be written as *F*_*CTn->CT50%,*_ where *n = 1…N*. The deformation vector fields were then applied to the dose maps to deform the doses onto the exhalation CT image and subsequently summed to obtain the delivered 4D dose in fraction *m*,

$$ {D}_{frac\_m}={\displaystyle \sum_{n=1\dots N}{F}_{C{T}_n->C{T}_{50\%}}{D}_{n,n0\_m}} $$

The above process was repeated for each fraction and the cumulative 4D dose through the SBRT treatment to the patient can be obtained by summing the fractional deformed dose and expressed as

$$ {D}_{tot}={\displaystyle \sum_{m=1,2,3,4}{\displaystyle \sum_{n=1\dots N}{F}_{C{T}_n->C{T}_{50\%}}{D}_{n,n0\_m}}} $$

#### D.2. Comparison between 3D planned and cumulative 4D doses

The dose statistics were compared between the initial 3D planned dose and the cumulative 4D dose calculated from the 4D-CT image sets. The PTV in the original 3D plans was generated from the ITV. The PTV defined on the 4D dose calculations was generated directly from the expansion of the GTV in the maximum exhalation phase of the 4D CT by the same physician. In the 4D dose calculation, GTV motion was already taken into account by the deformable image registration and the modified 4D PTV represents the PTV in that phase of the breathing cycle with setup error margin only. This was done to isolate the interplay effect more clearly for dosimetric comparison. If the original PTV, which included the ITV, was warped into a single phase, the coverage of the warped PTV will decrease even if there were no inter-play effects because dose warping was equivalent to evaluation in a frame of reference with no motion. The same set of organ at risks was also re-contoured by the same physician in the maximum exhalation phase of 4D-CT.

#### D.3. 4D Inter-fraction and cumulative inter-play effects

Inter-fraction effects were simulated using a different initialization phase of the 4D CT in the 4D dose calculation. This effect was evaluated by comparing the variation in dose (maximum difference minus minimum difference between fractions) as well as cumulative 4D doses with that calculated on the reference CT. The three patients that showed the largest difference in PTV coverage from the dynamic simulation were selected for further analysis. Histograms of the maximum dose difference per fraction from all four fractions within each voxel were generated for the PTVs and GTVs of the three patients. In addition, histograms of the cumulative dose difference between the cumulative 4D dose and the cumulative dose without motion calculated on the reference CT were generated for analysis. The cumulative dose without motion refers to the planned four fraction dose calculated on a single expiration phase CT. The impact of the motion on the dose distribution can be observed from the difference between the cumulative 4D dose and the cumulative dose without motion.

### Validation of plan partitioning with machine Dynalog files

This study in dynamic simulation utilized the control points defined by the patient treatment plan and the corresponding machine delivery timestamps recorded by machine delivery Dynalog files. In order to ensure that the plans were partitioned correctly with respect to the machine control point execution time, verification of the real time MLC position as recorded on the Dynalog files with the plan control points was performed.

## Results

### Difference between 3D planned dose and cumulative 4D dose

Differences in dosimetric parameters of the target and the OARs between the initial 3D planned dose and cumulative 4D dose using the constraints from RTOG 0915 were presented in the second column of Tables 1 and 2 respectively. V_105%_ of the PTV (V_x_ represents the percentage volume in the organ or target that receives more than x% of the prescribed dose) was on average about 5% smaller in the cumulative 4D doses. There was less than 1.5% difference in PTV V_95%_, V_90%_. The change in the ratios of prescription isodose volumes V__Presp. Iso_ and V__50%Presp. Iso_ to PTV volume V__PTV_ for all patients were less than 1.00. The intermediate dose spillage represented by D_2cm_ (the maximum dose 2 cm away from the PTV volume) was less than 2.2 Gy in all cases. For all OARs assessed doses, the mean differences in dosimetric parameters were less than 1.0 Gy. The dose constraints for all 15 patients, as specified in RTOG 0915, were maintained in the cumulative 4D dose.

**Table 1:** **Effect of motion on tumor target dose distribution in fifteen patients**

**Constraints**	**Difference between 3D planned dose and cumulative 4D doses Mean (min, max)**	**4D inter-fraction variation Mean (min, max)**	**Difference between cumulative 4D doses with and without motion Mean (min, max)**
PTV V_90%_	−0.48% (−1.44%, 0.25%)	0.20% (0.00%,1.89%)	−0.35% (−1.22%, 0.83%)
PTV V_95%_	−0.10% (−1.22%, 1.31%)	0.12% (0.00%, 1.38%)	−0.26% (−1.06%, 1.97%)
PTV V_105%_	−5.04% (−13.88%, 9.71%)	1.66% (0.00%, 6.87%)	2.75% (−5.90%, 4.65%)
V__Presp. Iso._/V__PTV_	−0.15 (−0.31, −0.01)	0.04 (0.00,0.15)	−0.30 (−0.91, −0.01)
V__50% Pres. Iso._/V__PTV_	−0.14 (−0.68, 0.06)	0.05 (0.00, 0.24)	−0.32 (−1.63, 0.00)
D_2cm_	−0.22 Gy (−2.20 Gy, 1.70 Gy)	0.45 Gy (0 Gy, 1.20 Gy)	−1.14 Gy (−4.00 Gy, 0.10 Gy)

The mean values are cumulative dose differences averaged over all patients. The mean values of the 4D inter-fraction variation are the average of the largest variations among four fractions over all patients.

**Table 2:** **Motion effect on OAR dose distribution in fifteen patients**

**Constraints**	**Difference between 3D planned dose and cumulative 4D doses Mean (min, max)**	**4D inter-fraction variation Mean (min, max)**	**Difference between cumulative 4D doses with and without motion Mean (min, max)**
**Spinal cord**			
**D**_**max**_	−0.03 Gy (−0.40 Gy, 0.20 Gy)	−0.05 Gy (0.00 Gy, 0.40 Gy)	−0.03 Gy (−0.30 Gy, 0.50 Gy)
**D_**_**0.35cc**_	0.00 Gy (−0.16 Gy, 0.17 Gy)	0.02 Gy (0.00 Gy, 0.05 Gy)	0.03 Gy (−0.12 Gy, 0.24 Gy)
**D_**_**1.2cc**_	0.01 Gy (−0.12 Gy, 0.14 Gy)	0.02 Gy (0.00 Gy, 0.06 Gy)	0.03 Gy (−0.13 Gy, 0.18 Gy)
**Esophagus**			
**D**_**max**_	−0.22 Gy (−1.30 Gy, 0.30 Gy)	−0.19 Gy (0.00 Gy, 0.80 Gy)	−0.37 Gy(−2.60 Gy, 0.30 Gy)
**D_**_**5cc**_	−0.06 Gy (−0.83 Gy, 1.37 Gy)	0.07 Gy (0.01 Gy, 0.21 Gy)	−0.16 Gy (−0.89 Gy, 0.30 Gy)
**Heart**			
**D**_**max**_	−0.51 Gy (−2.00 Gy, 0.90 Gy)	0.27 Gy (0.00 Gy, 0.80 Gy)	−0.63 Gy (−2.5 Gy, 0.90 Gy)
**D_**_**15cc**_	−0.18 Gy (−0.69 Gy, 0.92 Gy)	0.08 Gy (0.00 Gy, 0.23 Gy)	−0.38 Gy (−1.54 Gy, 0.33 Gy)
**Great vessels**			
**D**_**max**_	−0.52 Gy (−1.80 Gy, 6.00 Gy)	0.19 Gy (0.00 Gy, 0.40 Gy)	0.39 Gy (−1.10 Gy, 2.00 Gy)
**D_**_**10cc**_	−0.12 Gy (−2.44 Gy, 2.66 Gy)	0.04 Gy (0.00 Gy, 0.13 Gy)	−0.03 Gy (−1.05 Gy, 0.63 Gy)
**Trachea**			
**D**_**max**_	0.04 Gy (−2.10 Gy, 1.40 Gy)	0.03 Gy (0.00 Gy, 0.40 Gy)	0.13 Gy (0.00 Gy, 1.00 Gy)
**D_**_**10cc**_	−0.01 Gy (−0.42 Gy, 0.19 Gy)	0.03 Gy (0.00 Gy, 0.37 Gy)	0.01 Gy (−0.08 Gy, 0.14 Gy)
**Bronchus**			
**D**_**max**_	0.25 Gy (−2.00 Gy, 4.90 Gy)	0.37 Gy (0.00 Gy, 1.20 Gy)	0.75 Gy (−2.00 Gy, 7.80 Gy)
**D_**_**10cc**_	−0.42 Gy (−4.10 Gy, 2.98 Gy)	0.08 Gy (0.00 Gy, 0.25 Gy)	0.30 Gy (−0.40 Gy, 1.59 Gy)
**Rib**			
**D**_**max**_	−0.58 Gy (−3.00 Gy, 1.50 Gy)	0.29 Gy (0.00 Gy, 1.20 Gy)	−0.54 Gy (−1.50 Gy, 0.60 Gy)
**D_**_**1cc**_	−0.19 Gy (−1.58 Gy, 4.64 Gy)	0.10 Gy (0.01 Gy, 0.32 Gy)	−0.33 Gy (−0.99 Gy, 0.44 Gy)
**Total lung**			
**V_**_**20Gy**_	0.02% (−0.87%, 0.66%)	0.02% (0.00%, 0.14%)	−0.08% (−0.41% 0.15%)
**D_**_**1500cc**_	−0.03 Gy (−0.23 Gy, 0.64 Gy)	0.01 Gy (0.00 Gy, 0.09 Gy)	0.03 Gy (−0.13 Gy, 0.74 Gy)
**D_**_**1000cc**_	−0.03 Gy (−0.18 Gy, 0.16 Gy)	0.00 Gy (0.00 Gy, 0.02 Gy)	0.03 Gy (−0.09 Gy, 0.46 Gy)

The mean value are cumulative dose differences averaged over all patients. The mean values of the 4D inter-fraction variation are the average values of the largest variations among four fractions over all patients.

### 4D Inter-fraction and cumulative inter-play effect

**Figure 3:**
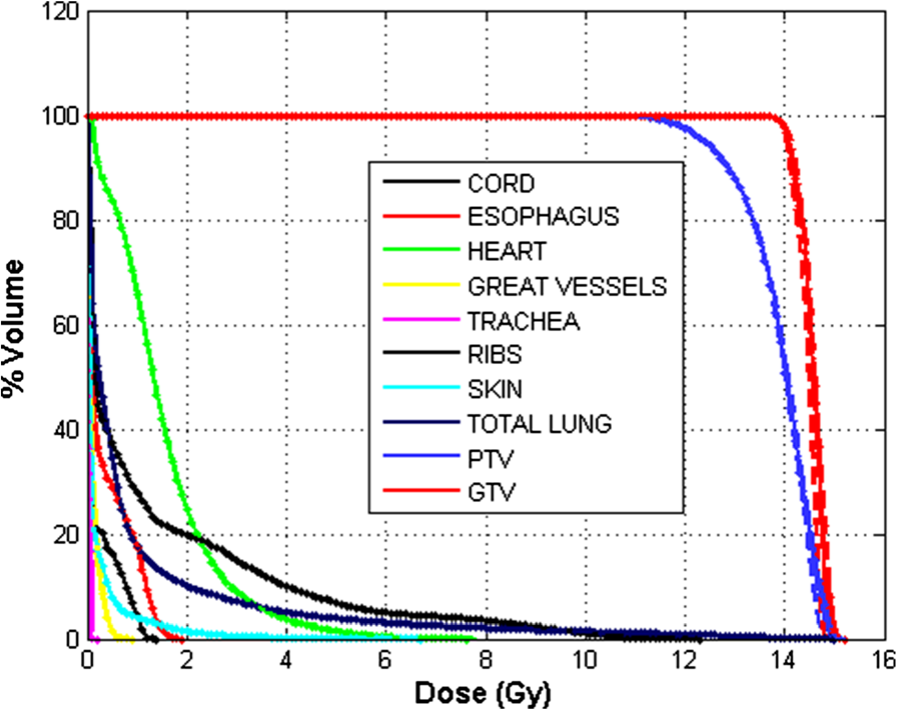
**The DVH comparison of the fractional dose for all four fractions when motion was considered.** Very limited differences in dose distributions were observed from the DVHs of four fractions.

Four single fraction DVHs of the dynamic delivery for one representative patient were plotted in Figure 3. There was very little variation between the DVHs for both the targets and OARs. Inter-fraction dose variation is summarized in column 2 of Tables 1 and 2 for all 15 patients. A mean variation of less than 1.7% for the target and 0.4 Gy for all OARs were observed in the 4D inter-fraction variations for all patients. One patient had a 6.9% difference in PTV V_105%_ between fractions. The difference between cumulative doses with and without motion is summarized in column 3 of Tables 1 and 2. Mean differences of less than 2.8% for the targets and 0.8 Gy for the OARs were observed in all patients.

**Figure 4:**
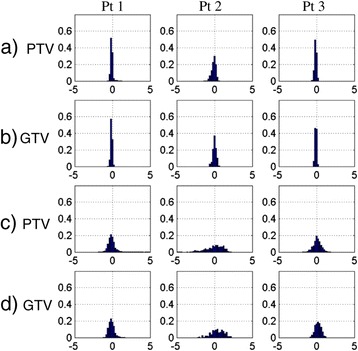
**Histogram representations of the motion effect. a)** and **b)** Inter-fraction interplay effect: the voxel histogram of the largest dose differences in PTV volume and GTV volume among all four fractions for three patients; **c)** and **d)** difference in cumulative 4D dose with and without motion: histogram of dose differences in the PTV and GTV due to motion. The bars represent the percentage of the voxels in the PTV or GTV with dose differences in 0.2 Gy bins.

**Figure 5:**
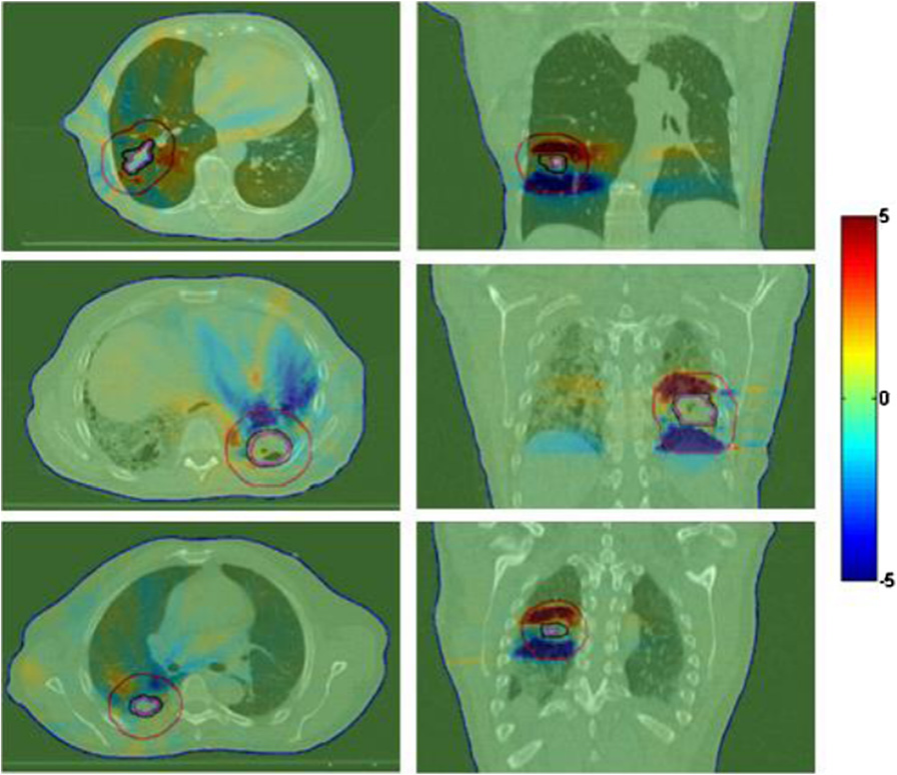
**Axial and coronal views of the dose difference with and without motion of three patients.** The colored contours denote the GTV (magenta), PTV (black), PTV + 2 cm (red) and body (blue). The color overlay of the dose difference range from −5.0 Gy to 5.0 Gy.

For the three patients selected for further analysis, the histograms of the largest dose difference per fraction in the PTV and GTV among all four fractions were presented in Figures 4a and 4b. The histograms of dose differences in the PTV and GTV between the cumulative 4D dose with and without motion were presented in Figures 4c and 4d. The latter histograms represented the cumulative dose difference and were wider than the fractional histograms. The spatial distribution of the dose differences was shown in Figure 5. Note that the largest effect due to motion appears at the superior and inferior borders outside the PTV, whereas much less dose difference was observed within the PTV.

### Verification of MLC control points with real-time Dynalog files

**Figure 6:**
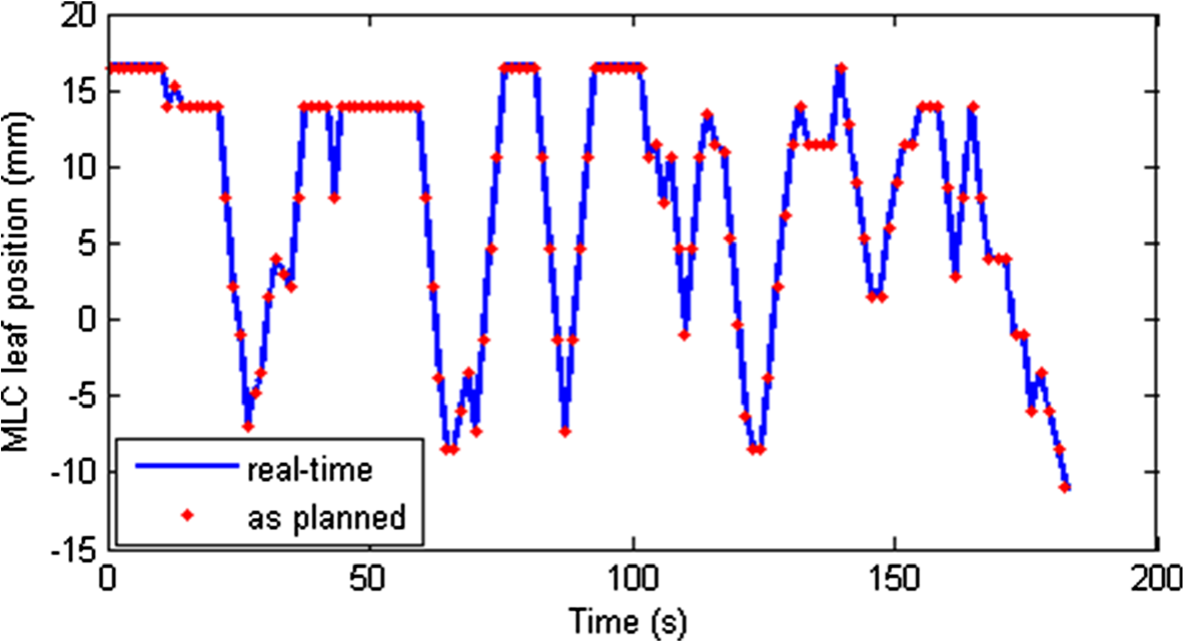
**Verification plot to show that the planned MLC leaf positions at the control points correspond to the actual MLC leaf motion during delivery.** These data correspond to MLC leaf 32 of bank A.

Examination of the machine delivery log files revealed that the delivery time varied from 127 to 185 seconds. The maximum dose rate was 600 MU/min but the mean dose rate was decreased to about 583 MU/min due to MLC motion speed limits. Figure 6 showed the positions of one MLC leaf that shadowed the tumor during plan delivery. The MLC position corresponded well with the planned MLC positions at the control points in real time. The maximum deviation of the MLC leaves from planned control points was 1.0 mm and the standard deviation was 0.23 mm. Such small deviations between the planned control points and delivery control points validated the above method of using the plan MLC leaf position in the dynamic simulation.

## Discussion

The above simulation results demonstrated that the effect of motion during the delivery of SBRT using RapidArc has limited effect on the target dose coverage, intermediate dose spillage and OARs, while a larger deviation was observed at the very high dose regions within the PTV. This could be related to the dose averaging effect due to tumor motion as others have observed [8]. Note that even for a single fraction, the dose deviation was small. This may be attributed to the fact that the dose per fraction was large in lung SBRT which in this case was more than six times the conventional dose per fraction (12.5 Gy vs. 1.8 to 2.0 Gy). The larger dose per fraction translated to longer treatment times and therefore more respiratory cycles to smooth out the dose during the delivery of each fraction. When all fractions were considered, it was observed that the effect of motion on the dose distribution was concentrated in the superior and inferior borders outside the PTV as shown in Figure 5. This was because the superior and inferior regions outside the PTV were in the radiation field at only some phases of the breathing cycle in the 4D analysis. When the maximum exhalation phase was chosen as the reference image, the superior border outside the PTV did not receive dose in the static plan but instead accumulates dose from the other phases when motion was considered. For early stage peripheral lung lesions, OARs such as the heart and bronchus were not located proximal to the target and therefore the impact of motion on the dose was small. The deviations in dose to the OARs were all smaller than 1.0 Gy in the dynamic simulation. However, in patients where the target was proximal to the critical organs, a larger heart and/or bronchus dose variation was observed.

We also verified that the MLC control points correlated well with the real time plan delivery with a small deviation in MLC positions. The impact from this small difference in actual delivery time on the dose distribution in this study was expected to be limited in this dynamic simulation and not clinically meaningful. However the 4D-CT image set used in this study was from the initial simulation CT, which might not represent the breathing pattern during the delivery. A more realistic dynamic simulation would require real-time respiratory monitoring or online fluoroscopy during patient treatment and is worth further study.

The results from this simulation study are in agreement with previous studies investigating the inter-play effect of VMAT lung SBRT [18,19]. While all previous studies as well as this one confirm that the inter-play effect can be considered small or negligible as far as target coverage is concerned, this study adds to the literature with regards to the impact on OARs using simulated patient data. The heart, great vessels and bronchus exhibited deviations of up to 2 Gy, 4.9 Gy and 6 Gy respectively due to respiratory induced motion into the target region that is not apparent when reviewing an initial plan calculated on the average CT image set. While these OAR doses remained well within the normal tissue constraints for the patients used in this study, motion into the target region should be factored into when treating a target located proximal to these structures.

One common assumption made in these studies is that the respiratory motion can be considered regular and reproducible during treatment delivery. There are two main sources of error with regards to an ITV based approach that can impact lung SBRT. The first error arises from irregular respiratory motion in 4D-CT which results in image artifacts and therefore impacts the accuracy of target definition [23]. The second error is the accuracy of the target motion envelope from a single 4D-CT scan. If a patient assumes a deeper inspiration at the time of treatment compared to the 4D-CT scan at the simulation, the target coverage may be compromised during SBRT delivery. This underlines the importance of pre-treatment verification using fiducials, cone beam CT or other image guidance technology in lung SBRT treatment delivery.

## Conclusions

This study investigated the effect of motion on IMAT SBRT plans using patient data. A detailed study of the inter-fraction and overall motion effect on PTV and OAR dose distributions was performed with the planned and simulated 4D dose. PTV coverage showed negligible effect due to motion while high dose spillage showed larger variation from perceived planned 3D dose. Limited motion effect was observed on OAR dose distributions except when the target volume was located near critical organs such as heart and/or bronchus. As IMAT SBRT is commonly employed in clinical practice, the results from this study are encouraging in confirming adequate dose distribution during patient treatment delivery at least for the patients who exhibit consistent regular breathing patterns.
